# A Performance Study of Deep Neural Network Representations of Interpretable ML on Edge Devices with AI Accelerators

**DOI:** 10.3390/s25185681

**Published:** 2025-09-11

**Authors:** Julian Schauer, Payman Goodarzi, Jannis Morsch, Andreas Schütze

**Affiliations:** Lab for Measurement Technology, Saarland University, 66123 Saarbrücken, Germany

**Keywords:** edge computing, smart sensors, interpretable ML, AI accelerator, latency, energy efficiency

## Abstract

With the rising adoption of machine learning (ML) and deep learning (DL) applications, the demand for deploying these algorithms closer to sensors has grown significantly, particularly in sensor-driven use cases such as predictive maintenance (PM) and condition monitoring (CM). This study investigated a novel application-oriented approach to representing interpretable ML inference as deep neural networks (DNNs) regarding the latency and energy efficiency on the edge, to tackle the problem of inefficient, high-effort, and uninterpretable-implementation ML algorithms. For this purpose, the interpretable deep neural network representation (IDNNRep) was integrated into an open-source interpretable ML toolbox to demonstrate the inference time and energy efficiency improvements. The goal of this work was to enable the utilization of generic artificial intelligence (AI) accelerators for interpretable ML algorithms to achieve efficient inference on edge hardware in smart sensor applications. This novel approach was applied to one regression and one classification task from the field of PM and validated by implementing the inference on the neural processing unit (NPU) of the QXSP-ML81 Single-Board Computer and the tensor processing unit (TPU) of the Google Coral. Different quantization levels of the implementation were tested against common Python and C++ implementations. The novel implementation reduced the inference time by up to 80% and the mean energy consumption by up to 76% at the lowest precision with only a 0.4% loss of accuracy compared to the C++ implementation. With the successful utilization of generic AI accelerators, the performance was further improved with a 94% reduction for both the inference time and the mean energy consumption.

## 1. Introduction

**Figure 1:**
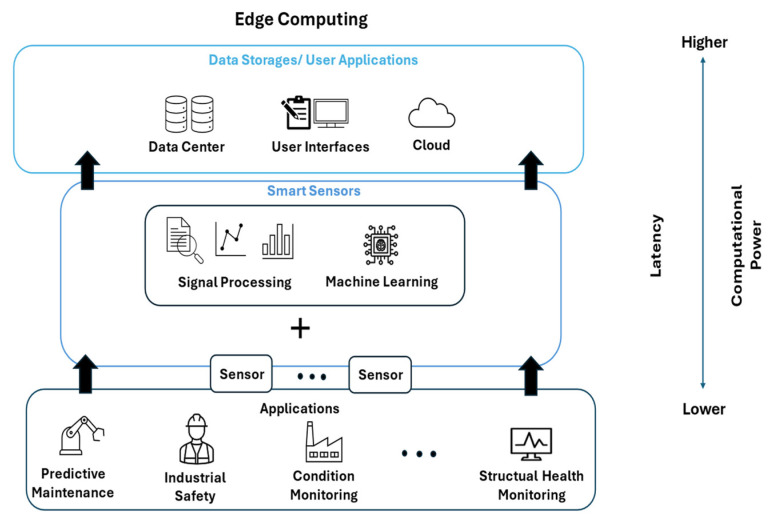
Edge computing workflow for reducing energy and latency.

In recent years, the number of machine learning (ML) applications has increased rapidly. The area of industrial application ranges from predictive maintenance (PM) [1] over structural health monitoring (SHM) [2] to different applications in the field of condition monitoring (CM) [3]. The widespread adoption of ML techniques across various processes has increased both the number of deployed sensors and the volume of collected data. To handle the massive amount of data, the focus has shifted from sending the collected data to centralized units with a high computational power and resources to smart sensors processing data directly on the edge. On-sensor pre-processing reduces the energy consumed by transmitting data to central units [4]. To extend this approach, training and inference processes can be executed in two different environments. The resource-intensive training process can be executed in a computationally powerful cloud system and the inference can be run directly on the operating sensor. By taking this approach, not only is the data transmission energy reduced, but the latency, a crucial factor in real-time applications, is also minimized (Figure 1).

These advantages regarding energy consumption and latency lead to a strong desire to implement ML algorithm inference directly onto sensors. Sensors that combine data collection and further data processing are referred to as smart sensors [5,6]. In addition to energy constraints, most smart sensor platforms have a limited storage capacity and computational power. In particular, complex ML models are computationally expensive and often need powerful and optimized hardware for them to run efficiently. This represents a significant challenge for implementing edge AI [7]. Edge computing and edge AI enhance the process of data processing and the following data transmission by providing computing resources directly on or near the sensors and the data collection system.

To tackle this problem, many IC and hardware manufacturers are developing hardware dedicated for ML algorithms, particularly neural networks (NNs) [8]. Most of these dedicated AI accelerators are based on application-specific integrated circuits (ASICs) or field-programmable gate arrays (FPGAs) to optimize the data processing directly on the edge [9,10,11]. Combined with available DL frameworks, like TensorFlow, Keras, or ONNX [12], and the corresponding DL compilers, this development empowers the efficient execution of DNNs on dedicated hardware. These frameworks allow for the training and also the inference on tensor processing units (TPUs), neural processing units (NPUs), and graphical processing units (GPUs) instead of general-purpose hardware like central processing units (CPUs), with minimal additional effort in hardware-specific programming [13,14,15].

These generic AI accelerators often enable computation capabilities through ML interfaces and frameworks that are best suited for usage with neural networks (NNs), especially DNNs [16,17,18]. However, DNNs are often criticized for producing uninterpretable results and exhibiting black-box behavior [19]. Conventional ML algorithms based on feature extraction (FE), feature selection (FS), and classification or regression (C/R) have gained attention [20,21,22] because of their robustness and interpretable properties [23]. Compared to DL approaches, these FESC/R algorithms comprising FE, FS, and C/R allow the user to analyze the data and features in each processing step of the ML pipeline freely, offering the physical interpretability of cause and effect. This enables a detailed analysis of the behavior of the ML system and facilitates the interpretation of its outcomes, thereby enhancing the transparency and reliability of the ML approach. This aspect is of particular importance for safety-critical applications and industrial environments.

One disadvantage of these interpretable ML algorithms is an inefficient or high-effort implementation on sensors or near sensors, as resource-constrained hardware often limit their usage in edge AI. While most of the recent studies [24,25] have focused on the inference of DNNs on the edge, investigations on and the usage of interpretable ML algorithms are comparably low. Other studies have tried to enable DNN accelerators for non-ML-based applications by leveraging the hardware [26], which requires a high-effort programming and modification step. A different approach is introduced in [27]. This approach tries to accelerate parts of a signal-processing pipeline on ASICs and FPGAs, but also leads to high-effort, hardware-specific programming.

We recently introduced the interpretable deep neural network representation (IDNNRep) to implement the inference of interpretable ML algorithms as DNNs [28,29,30]. This approach allows them to be run on typical AI accelerators, reducing the inference time and energy consumption without any further programming effort. This application-oriented method breaks down the inference of interpretable ML algorithms into basic mathematical matrix operations to represent them with DNN layers to enable generic AI accelerators for those algorithms. The fast matrix algebra allows the user to efficiently implement the inference on dedicated hardware, which greatly accelerates the matrix and vector operations. With the IDNNRep, the performance of interpretable ML algorithms is significantly improved and outperforms traditional computing architectures in terms of energy consumption and the inference time. This approach combines the interpretable characteristics of FESC/R algorithms and the efficient implementation of DNNs on dedicated hardware.

This study investigated the implementation of the novel DNN representation of interpretable ML algorithms on generic AI accelerators. A comparison of the accuracy, inference time, and energy consumption of the different implementations was benchmarked on two system-on-chip (SoC) hardware systems, including an NPU or a TPU. These metrics represent the most crucial figures of merit in edge computing. For this purpose, a detailed benchmark was executed for one regression and one classification task to compare the different implementations. The benchmark compared a Python code implementation, a C++ 17 code implementation, and different quantized IDNNReps executed in a Python 3.12 environment for both tasks regarding the accuracy, energy consumption, current, and inference time. Additionally, all the different implementations were benchmarked on both hardware systems. The IDNNRep was compared for three different precision levels from Floating Points 32 (FP32) and 16 (FP16) to Integer 8 (INT8), based on post-training quantization (PTQ) [31]. The energy consumption was measured using a hardware-level approach, which measured the power of the board and calculated the energy consumption during the inference time.

To address the above-mentioned problems, this paper makes the following key contributions:

- We introduced a novel method to run interpretable ML on DNN-specific AI accelerators.
- The proposed method outperforms conventional implementations in terms of the inference time and energy efficiency.
- The proposed method was evaluated using implementations at different quantization levels on two hardware platforms.

The rest of this paper is structured as follows: Section 2 describes the materials and methods used in this study, including a short explanation of the interpretable ML approach and the used ML toolbox. Additionally, Section 2 briefly describes the methodology of the IDNNRep, the DL frameworks and used hardware, the dataset, and the measurement setup. Section 3 presents the results of the measurements on the different edge hardware, including the dedicated AI accelerators. Section 4 discusses the results of the different implementations, and Section 5 concludes the paper and gives an outlook on future work.

## 2. Materials and Methods

### 2.1. Interpretable Machine Learning Approach and ML Toolbox

This paper focuses on implementing the inference of interpretable ML efficiently on edge hardware. One approach of interpretable ML is to design a workflow that relies on the input signal’s physically interpretable features. For this purpose, an algorithm consisting of FE, FS, and C/R is created (FESC/R). The open-source interpretable ML toolbox [32], implemented in MATLAB R2023b [33], provides different complementary methods. The demonstration of the functionality of the DNN representation method is based on parts of the ML toolbox and contains four FE, four FS, and two C/R algorithms.

Additionally, the toolbox provides an automated ML model selection based on a benchmark comparison of all the available combinations in the toolbox. This allows for the selection of the best available interpretable ML model for a specific dataset. The ML toolbox, which forms the basis of the DNN representation in this paper, contains the methods listed in Table 1.

#### 2.1.1. Feature Extraction

FE algorithms aim to reduce dimensionality by extracting the input data’s physically meaningful, interpretable features. Different FE algorithms extract features from various domains, allowing developers to use physical or system-specific prior knowledge to extract useful information. Reducing and representing input data always involves a trade-off, which involves keeping the number of features low while maximizing information value in the features. Some of the used FE methods rely on a prior training process to determine the internal parameters, while others are non-trainable and can be applied directly without any learning phase.

#### 2.1.2. Feature Selection

FS methods are a supervised component of the machine learning workflow. They aim to identify the most important features produced during the previous FE step. FS techniques evaluate and rank the extracted features based on their informational value, to reduce the data size while retaining as much information as possible.

#### 2.1.3. Classification/Regression

The final component of the FESC/R pipeline is either a classification or regression algorithm. This element is also a supervised and trainable part of the workflow, responsible for generating the final output. Classification algorithms aim to map the input data to predefined categorical outputs while minimizing the error rate. In contrast, regression algorithms are used to predict numerical values, typically aiming to minimize metrics such as the root mean squared error (RMSE).

### 2.2. Interpretable Deep Neural Network Representation

**Figure 2:**
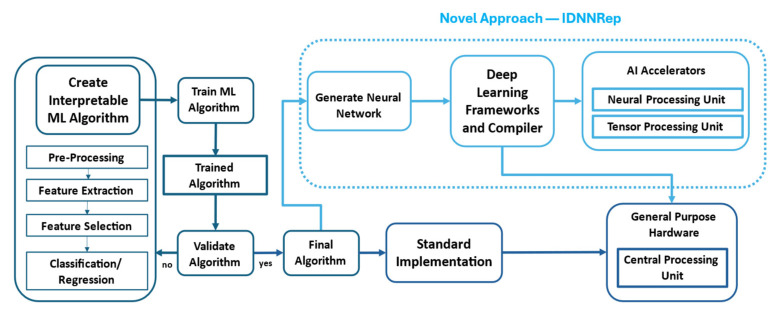
Flowchart of the IDNNRep used to enable generic AI accelerators for interpretable ML. This study investigated the difference in the implementation of the IDNNRep on a standard CPU compared to NPU/TPU processors regarding the energy consumption and inference time.

The core element of this study was the novel approach used to represent the inference of the trained interpretable ML algorithm as DNNs, the IDNNRep. This section briefly describes the proposed method; a detailed description of the method is published in [29]. To find an appropriate ML algorithm consisting of an FS, FE, and regression or classification method, the AutoML toolbox [20] allows for the training and testing of all the available combinations in the pool of methods listed in Table 1. This phase includes the validation of each ML algorithm (see Figure 2), typically using 10-fold cross-validation or leave-one-group-out CV [23]. After the appropriate ML algorithm has been identified, the static inference of the algorithm can be implemented, for example, by using Python or preferably C++ code on general-purpose hardware. An alternative approach involves implementing the inference on FPGAs or ASICs to achieve hardware-level acceleration, at the cost of a significantly more complex and resource-intensive development process. This study evaluated the IDNNRep to enable generic AI accelerators such as an NPU or TPU. These accelerators were implemented as ASICs optimized for processing DNN layers, with their primary benefits being a reduced inference time and an improved energy efficiency. The hardware was developed to enable and optimize AI inference on the edge, directly within the data acquisition system.

**Figure 3:**
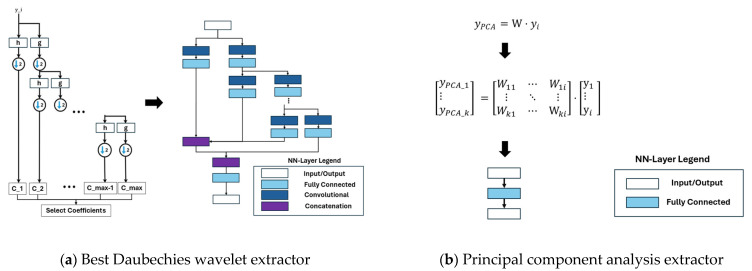
(**a**) IDNNRep of the best Daubechies wavelet extractor; (**b**) IDNNRep of the principal component analysis extractor.

The IDNNRep breaks the static inference operations of the interpretable ML algorithm down into basic mathematical operations. These mathematical operations, including summation, division, multiplication, square root calculations, and filtering, can be efficiently implemented using standard DNN layers. After the initial training of the FESC/R algorithm, no further modelling is required. To convert the inference successfully to an efficient hardware-executable format, an in-depth knowledge of the to-be-implemented FESC/R algorithms is necessary. Using this approach, the execution of the inference process of an interpretable ML algorithm can be seen as a static computational graph that sequentially performs mathematical operations. As an example of the IDNNRep, Figure 3 illustrates the DNN representations of the BDW and PCA feature extraction.

The implementation of the full AutoML toolbox is described in detail in a previous study [29]. To create the inference of a complete FESC/R algorithm, individual computational graphs were concatenated, enabling the dynamic integration of the methods. In this paper, the IDNNRep was applied to the open-source AutoML toolbox [32]; however, it is generalizable to arbitrary mathematical and statistically based algorithms. Once the conversion is complete, additional benefits of DNNs become available beyond the efficient implementation on edge hardware, including transfer learning (TL) techniques, which are typically not applicable to conventional ML algorithms.

### 2.3. Deep Learning Frameworks

Due to the wide use of DL, a wide range of deep learning frameworks (DLFs) are available. Two of the most common frameworks are the Open Neural Network Exchange (ONNX) [44] and TensorFlow [45]. The benefit of these two DLFs is the interoperability due to easy conversion methods between both frameworks. Both deliver runtime versions for the model inference on the edge, named ONNX Runtime and TF Lite, which allow for efficient implementation on edge hardware.

Additionally, common converters enable the quantization [46,47] of the DNN from FP32 to FP16 and INT8 [48]. The quantization allows for an investigation of implementations of the DNN with different levels of precision. Since most AI accelerators, including the hardware used in this study, only support quantized INT8 DNNs, the process of quantization is a prerequisite. In TensorFlow Lite, FP16 models are executed with native FP16 instructions on CPUs that support them, while on CPUs without full FP16 support, the computations are internally converted to FP32 and the results are cast back to FP16. In contrast, INT8 models utilize optimized integer kernels that leverage specialized hardware instructions. In this study, TensorFlow and TensorFlow Lite were used as DLFs. This decision was based on the fact that both hardware platforms tested in this study support the delegation of TensorFlow Lite operations to the specific AI accelerator. Post-training quantization (PTQ) was selected as a quantization technique [46]. This quantization method enabled the interpretability of the ML models to be maintained, which could be compromised by quantization-aware training. Quantization-aware training would retrain the weights of the IDNNRep to optimize the results. Retraining would alter the predefined architecture and weights of the IDNNRep networks, leading to changes in the extracted features and, consequently, in the interpretability based on those features.

### 2.4. Hardware

To demonstrate the generic usage of the novel approach with different AI accelerators, two different hardware platforms were chosen to demonstrate the functionality of the IDNNRep. The hardware platforms were two single-board computers based on the NXP iMX 8M Plus system-on-chip (SoC) [49]. The main difference between both platforms was the unique co-processors. The QSXP-ML81 included an NPU, while the Coral Dev Board [50] included a TPU. These AI co-processors are based on ASICs providing high-performance ML inferencing. This study compared the different implementations on two platforms. The following section briefly describes the specifications and functionalities of the two hardware platforms.

#### 2.4.1. NXP Neural Processing Unit

**Figure 4:**
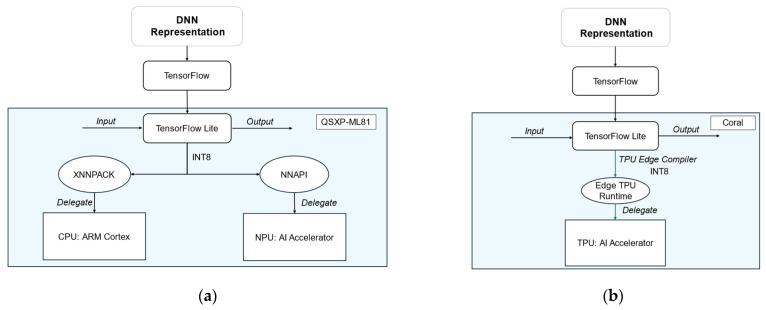
(**a**) Software stack of the neural processing unit on the QXSP-ML81; (**b**) software stack of the tensor processing unit on Coral.

To enable the NPU, the IDNNRep of the FESC/R algorithms was deployed as a TensorFlow Lite model on the QSXP-ML81. The model must be quantized to INT8 precision. The delegation of the TensorFlow Lite model can be performed in a Python script by creating an interpreter of the model that points to the NNAPI. The NNAPI then delegates the supported DNN operation of the DNN representation to the NPU (Figure 4a). The NNAPI executes the supported operations on the NPU and the unsupported operations on the CPU. Additionally, the QSXP-ML81 enables the efficient execution of the quantized INT8 DNNs using XNNPACK on the CPU of the I.MX8 M processor.

#### 2.4.2. Google Tensor Processing Unit

The Google Coral TPU supports TensorFlow Lite models, also within a Python script, which delegates the appropriate DNN operations to the TPU (Figure 4b). To provide a high-speed neural network performance, the edge TPU supports a specific set of DNN operations that can be executed on the TPU. An edge-compiled version of the model has to be created to run the model on the TPU.

### 2.5. Data

The data used are an example from the field of PM recorded on a hydraulic system (HS) equipped with multiple sensors [51]. The dataset consisted of four targets, which can be seen as separate problems. The goal was to detect various faults or degradations of the system. The system variables, the valve state and accumulator, were selected as the target variables for training the algorithms in this paper. Both the targets were treated as two independent datasets: HS (Acm) and HS (Valve). The work cycle of the pressure sensor (PS1) shows the best correlation with these targets. This allows a single dataset to be utilized for both a regression and a classification problem (Table 2).

### 2.6. Evaluation

**Figure 5:**
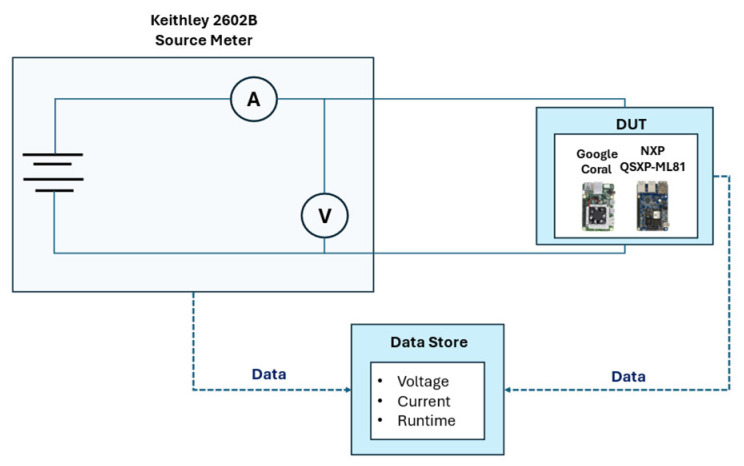
Testbench for inference time and energy consumption measurement.

The evaluation of the IDNNRep was based on several measurements, with the NXP QSXP-ML81 and Google Coral representing an application-specific analysis to allow for concrete statements in terms of the inference time and energy consumption. The measurement setup consisted of three main components: a Keithley 2602B Source Meter, the device under test (DUT), and a data store for the recorded values. Figure 5 describes the measurement setup used to measure the inference time, current, and energy consumption.

The Keithley 2602B Source Meter [52] is a highly accurate source measure unit (SMU) that combines a high-precision voltage or current source with a high-precision power measurement, allowing for an accurate measurement of the power consumption of the connected hardware. Compared to other studies [53,54], this represents a hardware-level approach that can be used to measure the actual consumption of connected edge hardware. The DUT was connected to the source meter with a USB-C connection. The source meter delivered a constant 5 V DC voltage to power the DUT, while the current consumed by the DUT was measured by the source meter. Table 3 lists the specifications for the current measurement and the voltage supply.

The measurement accuracies were incorporated into the energy evaluation via error propagation to ensure the validity of the measurement results. The specific design of the experiment for each measurement is described in the following sections. Except for the C++ implementation, all variants were executed using Python scripts. The DNN models were invoked through the TensorFlow and Tensor Flow Lite APIs available in Python, which also manage delegation to the AI accelerators [55].

#### 2.6.1. Accuracy

In addition to the hardware-related metrics on the edge, such as the inference time and energy consumption, the accuracy of the implemented interpretable ML algorithms represents a key evaluation metric, especially concerning the changing quantization levels of the underlying DNN representations. The accuracy of both the classification ${acc}_{class}$ and regression ${acc}_{reg}$ models was assessed as follows, respectively:

(1) $${acc}_{class}=1-\frac{1}{n}\sum_{i=1}^{n}\left[Y_{pred_{i}}\neq \;Y_{actual_{i}}\right],\;$$

(2) $${acc}_{reg}=1-\frac{\sqrt{\frac{1}{n}\sum_{i=1}^{n}{\left(Y_{pred_{i}}-Y_{actual_{i}}\right)}^{2}}}{max(Y_{actual})}$$

$Y_{pred}$ describes the prediction of the model and $Y_{actual}$ is the true target value. To validate the prediction of the interpretable ML algorithms, a 10-fold stratified cross-validation was performed. This validation approach divides the dataset into ten equally sized subsets while preserving the original class distribution across the folds. The model was trained on nine folds for each iteration and evaluated on the remaining one. After all ten folds had been used as test sets once, the overall accuracy was computed based on the aggregated predictions across all the test folds.

#### 2.6.2. Inference Time

The inference time comparison for the different implementations of the interpretable ML algorithms was based on two key metrics. The first metric was the mean inference time $\bar{t}$, measured using the high-resolution timing libraries in Python and C++ [56,57]. Each implementation was executed k = 10,000 times, where each inference time $t_{i}$ was recorded. After the 10,000 inferences, the mean inference time was calculated as follows:

(3) $$\bar{t}=\frac{1}{k}\cdot \sum_{i=1}^{k}t_{i}$$

In addition to the mean inference time, the standard deviation ∆t was calculated to show the relevance of the differences between the inference time measurement results. To ensure that the variations caused by initialization did not influence the results, the initialization phases were excluded from the measurement, so that the observed inference times followed a normal distribution $N(\bar{t},{∆t}^{2})$:

(4) $$∆t=\sqrt{\frac{1}{k-1}\cdot \sum_{i=1}^{k}{{(t}_{i}-\bar{t})}^{2}}$$

The resulting inference time is presented in a bar chart with the height of the mean and an additional error bar representing the standard deviation.

#### 2.6.3. Energy Consumption

**Figure 6:**
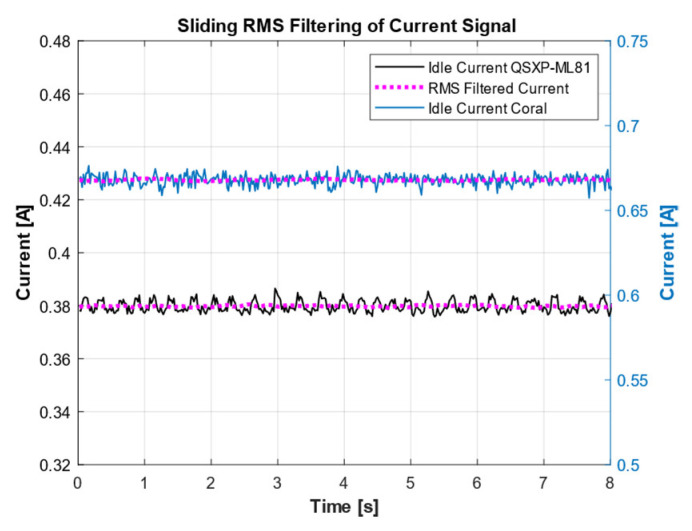
Example of RMS filtering on the current in idle mode for both boards.

Another metric under investigation was the energy consumption. The energy consumption was derived from the current and voltage measurements taken by the SMU. Both were recorded at a sampling rate of 50 Hz during the 10,000 inferences. A sliding RMS filter, with a window size of 100 samples, was applied to the current signal to improve the signal quality and reduce measurement noise (Figure 6).

The power $P(t_{i})$ was multiplied by the sample time $t_{s}$ and then summed over the total number of samples *n* to calculate the energy $W_{all}$ for all inferences:

(5) $$W_{all}=\sum_{i=1}^{n}P(t_{i})\cdot t_{s}=\sum_{i=1}^{n}U\cdot \;I\left(t_{i}\right)\cdot t_{s}$$

By summing up the product of the constant voltage U and the discrete currents $I\left(t_{i}\right)$ during the k inferences, the total electrical energy consumption over all the inferences was calculated. By dividing the total energy by the number of inferences k, an estimation for one inference $W_{mean}$ was achieved:

(6) $$W_{mean}=\frac{W_{all}}{k}$$

**Figure 7:**
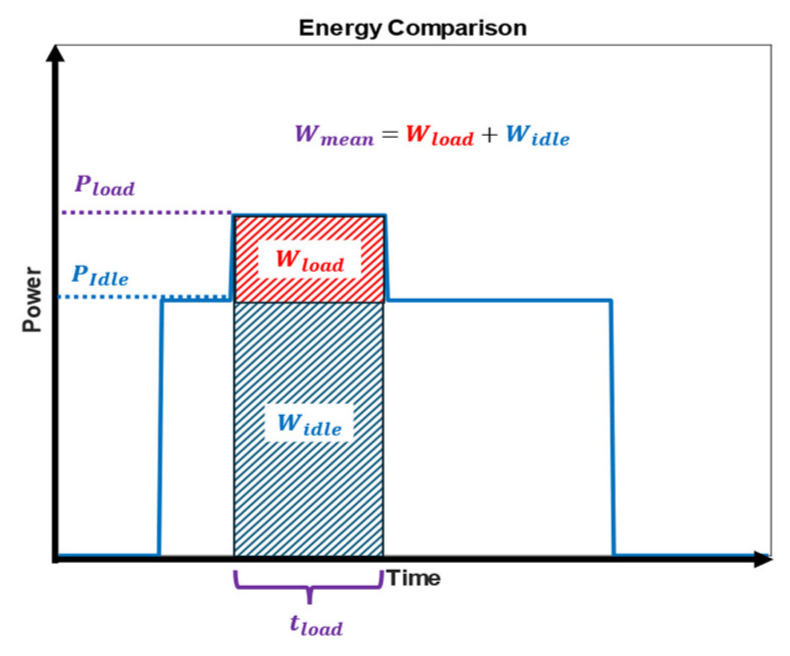
The difference between the mean energy and the load energy.

The mean energy $W_{mean}$ simulates an inference if the edge hardware switches between inference and a sleep mode with very low energy consumption. Another important energy-related metric is the difference between the mean energy consumption $W_{mean}$ and the energy consumed in idle mode $W_{idle}$ during the same inference time, namely the load energy $W_{load}$ (see Figure 7).

This metric simulates the energy consumption of edge devices if the device does not switch into sleep mode and remains in idle mode. The specific hardware in use largely determines the idle current $I_{idle}$. Beyond the absolute energy consumption, this metric evaluates the relative efficiency gain compared to the idle state. As shown in Figure 7 $W_{load}$, the difference between the mean energy consumption and the energy consumed during idle mode is presented.

(7) $$W_{load\_all}=W_{all}-W_{idle}=W_{all}-\sum_{i=1}^{n}U\cdot \;I_{idle}(t)\cdot t_{s}$$

By dividing the difference $W_{load\_all}$ by the number of inferences k, the average energy consumption per inference $W_{load}$ can be determined. This value was derived from the current measurements and was calculated as follows:

(8) $$W_{load}=\;\frac{W_{load\_all}}{k}\;$$

The uncertainties in the current measurement ∆I and the voltage source ∆U were considered via error propagation applied to the energy consumption $∆W_{el}$, enabling a comparison between the different implementations. This allowed for an analysis of the influence of the measurement uncertainty on the results. As the integer number of the inferences was free of uncertainty, the error propagation for the energy measurement was determined using Gaussian error propagation, which assumes that ∆U and ∆I are statistically independent:

(9) $$∆W_{el}=\sqrt{{\left(\frac{dW_{el}\;}{dU}\cdot \;∆U\right)}^{2}+{\left(\frac{dW_{el}\;}{dI}\cdot \;∆I\right)}^{2}}$$

(10) $$=\sqrt{\sum_{i=1}^{n}{\left(I\left(t_{i}\right)\cdot \;∆U\cdot t_{s}\right)}^{2}+{\left(U\cdot \;∆I\left(t_{i}\right)\cdot t_{s}\right)}^{2}}$$

∆U and ∆I describe the current measurement’s combined uncertainty based on the SMU specifications given in Table 3. The combined uncertainty consists of the relative uncertainty ${(∆I}_{rel},{∆U}_{rel})$ and the absolute uncertainty ${(∆I}_{abs},{∆U}_{abs})\;$ and is calculated as follows:

(11) $$∆I=\sqrt{{{(∆I}_{rel})}^{2}+{{(∆I}_{abs})}^{2}}$$

(12) $$∆U=\sqrt{{{(∆U}_{rel})}^{2}+{{(∆U}_{abs})}^{2}}$$

This error propagation is depicted by the error bars in the bar charts for the energy consumption shown in Section 3.

## 3. Results

This section presents the results of the regression and classification tasks performed on the selected edge hardware. First, the accuracy at each precision level and the resulting DNN representation for both tasks are presented. Subsequently, the measured inference time, current, and energy consumption are analyzed.

### 3.1. Interpretable ML Algorithm

As mentioned earlier, only the best possible combination of the algorithms in the implemented AutoML toolbox was converted into the DNN representation. These interpretable FESC/R algorithms formed the base of the later-created DNN representations. The regression stack for the HS (Acm) dataset consisted of the StatMom extractor, followed by RELIEFF to select 15 features and the PLSR regression. For the classification, the best stack consisted of the ALA extractor, the Pearson correlation to select the 10 highest-ranked features, and a final LDA-MD classification. The achieved accuracies are listed in Table 4.

**Figure 8:**
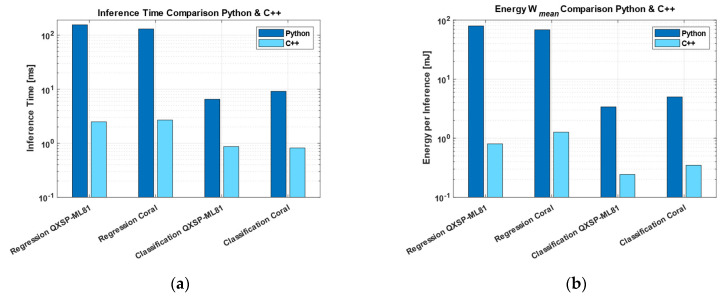
(**a**) Inference time comparison of the C++ and Python implementation; (**b**) energy comparison of the C++ and Python implementation.

Since the focus was more on the inference time and energy performance, the accuracy is briefly discussed in this section. Quantizing from FP32 to INT8 resulted in a 5.9% drop in the regression accuracy. Although this may seem like a substantial loss in accuracy, it represents only a 7.7 bar increase in the RMSE, which should be considered in relation to the target range (90–130 bar) to assess whether the prediction remains reasonable. For the classification accuracy, the reduction in precision from FP32 to INT8 resulted in a drop of only 0.5%. In a preliminary measurement, a first comparison between the Python and the C++ code implementations of the classification and regression algorithms determined that both showed huge differences, particularly concerning the inference time and the energy consumption. Figure 8 illustrates the significant gap in the performance between the C++ and Python implementations on a logarithmic scale. The pre-compilation of the C++ code allowed the CPU to execute instructions directly with minimal overhead. Python, as an interpreted language, introduces additional layers between the code and hardware, causing runtime overhead and a reduced execution efficiency. In the following comparison, C++ was therefore used as the baseline signal against which the IDNNRep was evaluated.

### 3.2. IDNNRep

#### 3.2.1. Regression Dataset HS (Acm)

**Figure 9:**
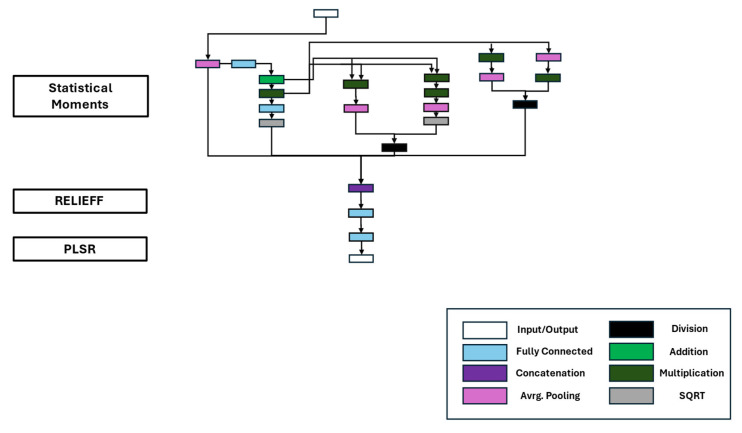
DNN representation of the interpretable ML algorithm for the regression task.

The complete regression network consisted of the concatenated networks of the StatMom, RELIEFF, and PLSR IDNNRep. The StatMom mainly consisted of basic mathematical operations like addition, division, multiplication, and square root layers. These layers were combined with average pooling and fully connected layers. The RELIEFF algorithm can be implemented with a single fully connected layer, as can the PLSR. The resulting DNN is shown in Figure 9. A detailed description of the resulting DNN representation is provided in [29].

#### 3.2.2. Classification Dataset HS (Valve)

**Figure 10:**
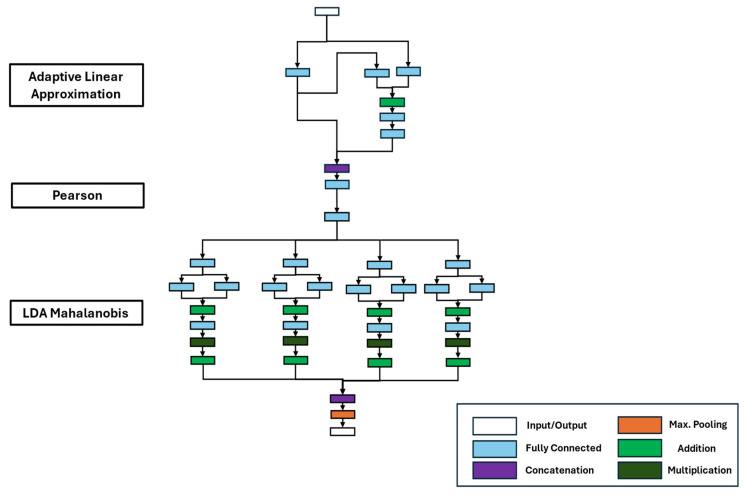
DNN representation of the interpretable ML algorithm for the classification task.

The classification network consisted of the representations of the ALA extractor, the Pearson selection, and the final LDA Mahalanobis classifier (see Figure 10). The ALA extractor comprised six fully connected layers, one addition layer, and one concatenation layer. The selection, similar to the regression network, was represented by one fully connected layer. The LDA-MD classifier consisted of four paths, representing the four target classes of the dataset. Each path calculated the LDA transformation and the corresponding Mahalanobis distance to each class center with four fully connected layers, two addition layers, and one multiplication layer. Afterward, the sign was changed, the calculated distances were combined, and the smallest distance was determined with a max. pooling layer.

### 3.3. Inference Time

**Figure 11:**
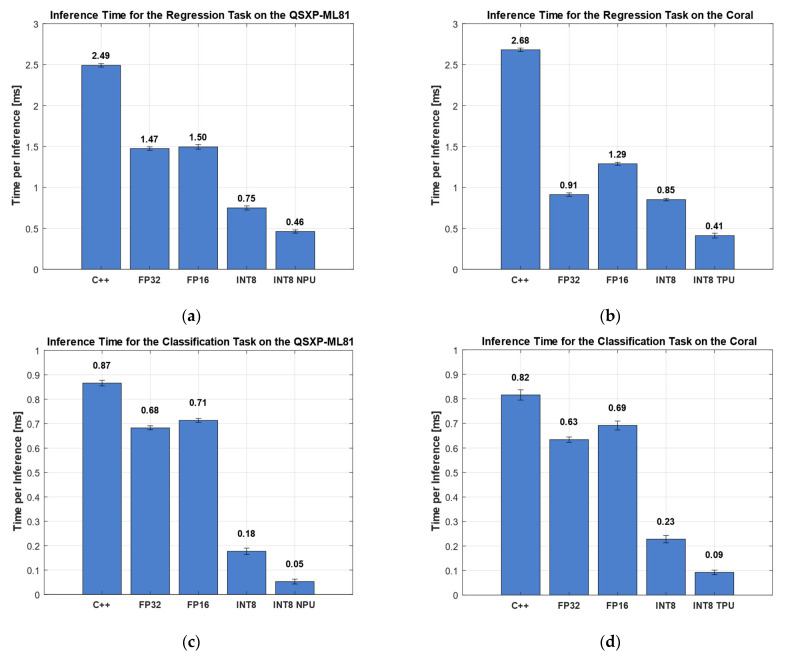
(**a**) Inference time of regression task on QXSP-ML81; (**b**) inference time of regression task on QXSP-ML81; (**c**) inference time of classification task on QXSP-ML81; and (**d**) inference time of classification task on Coral.

The inference time comparison in Figure 11 shows five implementations of the algorithms on the two hardware platforms tested, i.e., C++ FP32, FP16, INT8 on the CPU (ARM cortex), and INT8, using the respective AI accelerator.

#### 3.3.1. Regression Dataset HS (Acm)

On the QXSP-ML81 platform, the Python-based reference implementation showed the highest inference time, averaging 155.20 ms per inference (Figure 8a). In contrast, the low-level C++ implementation significantly reduced the inference time to 2.49 ms, representing a substantial performance improvement (Figure 11a). The FP32 and FP16 implementations yielded nearly identical inference times—1.47 and 1.50 ms, respectively—with overlapping standard deviations. INT8 quantization led to a further reduction, halving the inference time to 0.75 ms. The INT8 implementation executed on the NPU achieved the lowest inference time of only 0.46 ms per inference, a further improvement of xx% compared to the ARM cortex CPU.

The inference time measurements on the Coral board followed a similar trend to those observed on the QXSP-ML81 platform (Figure 11b). Again, the Python implementation exhibited the highest inference time, with 130.27 ms (Figure 8a), comparable to the Python inference time on the QXSP-ML81. The C++ implementation significantly reduced this to 2.68 ms. The FP32 DNN representation further improved the performance, reducing the inference time to 0.91 ms. Interestingly, the FP16 variant showed an inference time increase of 1.29 ms, making it less efficient than FP32 in this case. The INT8-quantized version led to an inference time of 0.85 ms, and the INT8 implementation delegated to the TPU achieved the lowest inference time at 0.41 ms, a reduction of more than 50%, compared to the INT8 version on the CPU.

#### 3.3.2. Classification Dataset HS (Valve)

The same analysis was performed for the classification task. On the QXSP-ML81, the Python implementation again showed the longest inference time, averaging 6.50 ms (Figure 8a). The C++ implementation reduced this to 0.87 ms. The FP32 model achieved an inference time of 0.68 ms, while the FP16 variant, similar to the regression task, performed slightly worse, with an inference time of 0.71 ms. Substantial improvements were observed with INT8 quantization: the INT8 implementation on the CPU achieved 0.18 ms, and further acceleration was achieved by the INT8 NPU version, which achieved the lowest observed inference time of only 0.05 ms, i.e., an improvement of more than 70% vs. the CPU and nearly 95% compared to the C++ reference implementation, with only a minimal loss of accuracy.

On the Coral board, the Python implementation also exhibited the highest inference time, at 9.12 ms. The C++ implementation significantly improved the performance, reducing the inference time to 0.82 ms. Additional reductions were achieved using DNN representations: the FP32 model lowered the inference time to 0.63 ms, while the FP16 version was again slightly higher, with 0.69 ms. A substantial performance gain was observed with the INT8-quantized model, achieving an inference time of 0.23 ms. The best performance was obtained with the INT8 model executed on Coral’s NPU, reducing the inference time to 0.09 ms, an improvement of 60% vs. the CPU and approx. 89% compared to the C++ reference implementation.

Table 5 presents the relative change in the inference time for the regression and classification tasks compared to the C++ implementation, which was seen as the baseline. The results highlight the performance improvements achieved using the different DNN representations, with the change in the percentage calculated relative to the C++ implementation. The table illustrates that each quantization level except FP16 led to a noticeable decrease in the inference time. The most significant improvements were observed when executing the interpretable ML models on dedicated AI accelerators, resulting in inference time reductions of up to 94% compared to the C++ baseline.

### 3.4. Current

**Figure 12:**
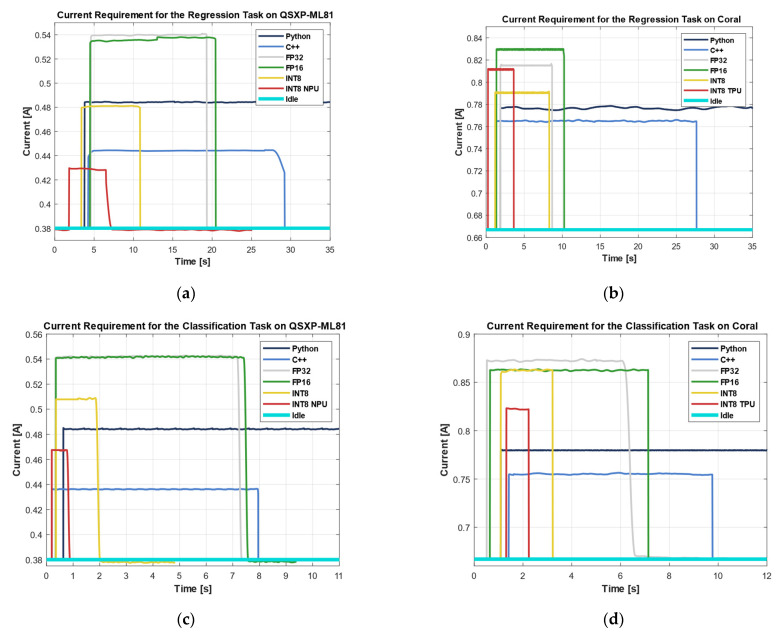
(**a**) Current of regression task on QXSP-ML81; (**b**) current of regression task on Coral; (**c**) current of classification task on QXSP-ML81; and (**d**) current of classification task on Coral.

Figure 12 depicts the SMU’s current measurements while executing 10,000 inferences at a constant voltage level. The QXSP-ML81 operated at an idle current of approx. 0.38 A (Figure 12a). The highest current during the regression tasks was observed for the FP32 and FP16 DNN implementations, each reaching approx. 0.54 A. The Python and INT8 implementations followed closely, with approx. 0.48 A. The inference time for the Python code can be seen in Figure 8, as Figure 12 does not show the completion of the task for this code. The C++ implementation showed a slightly lower current consumption, at 0.44 A. The lowest current was observed for the INT8 implementation executed on the NPU with approx. 0.43 A.

The Coral board showed a higher idle current consumption than the QXSP-ML81, drawing approx. 0.67 A. Among the regression tasks, the highest current was observed for the FP16 implementation of the DNN, reaching 0.83 A. The FP32 implementation and the INT8 variant executed on the TPU showed nearly identical current levels of around 0.82 A. A slightly lower current consumption was measured for the INT8 (CPU) and Python implementations, at 0.79 and 0.78 A, respectively. The lowest current was recorded for the C++ implementation, with approx. 0.76 A.

For the classification task on the QXSP-ML81, the highest current consumption was observed during the execution of the DNN FP32 and FP16 implementations, drawing approx. 0.54 A. The DNN INT8 implementation followed, with a current demand of 0.51 A, while the Python implementation consumed 0.48 A. The INT8 model executed on the NPU further reduced the current consumption to 0.46 A, slightly higher than the C++ implementation, which showed the lowest consumption at 0.44 A.

For the classification on the Coral board, the highest current was consumed by the DNN implementations executed on the CPU, with the DNN FP32 and DNN INT8 needing 0.86 A and the DNN FP16 being slightly higher, with 0.87 A. The DNN INT8 TPU implementation was slightly more efficient, with a demand of 0.83 A. The currents for the Python and C++ implementations were similar to the regression problem with 0.78 and 0.75 A, respectively.

### 3.5. Energy Consumption

**Figure 13:**
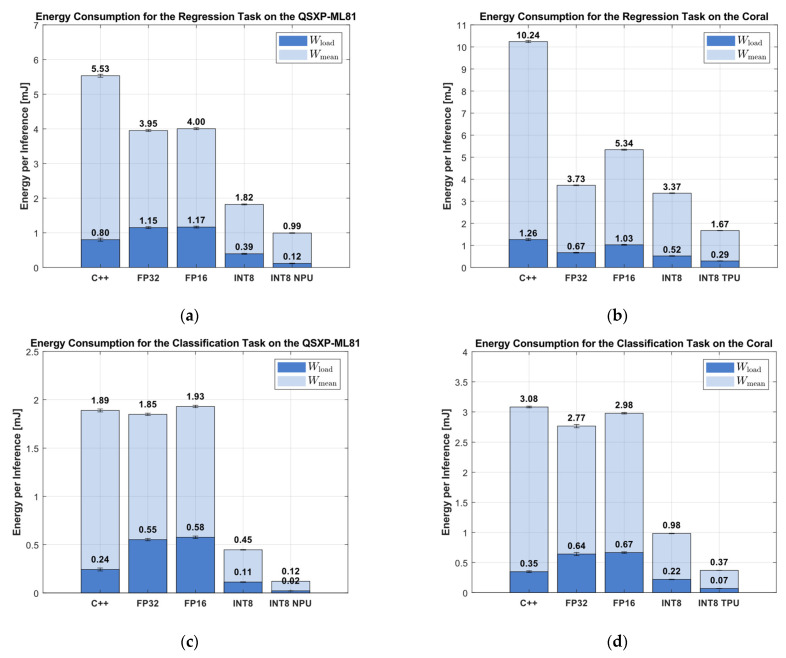
(**a**) Energy of regression task on QXSP-ML81; (**b**) energy of regression task on Coral; (**c**) energy of classification task on QXSP-ML81; and (**d**) energy of classification task on Coral. The bar graphs show both the total energy consumption $W_{mean}$ and the difference to the idle state $W_{load}$.

The energy consumption metric reflects the combined effects of the inference time and the current requirement to execute the inference, and is shown in Figure 13.

Among all the implementations, the Python version was the most energy-inefficient for the regression tasks, with a mean consumption of 374 mJ per inference (see Figure 8) on the QXSP-ML81 and 505 mJ on the Coral board. The C++ implementation significantly reduced the mean energy consumption to 5.53 mJ (QXSP-ML81) and 10.24 mJ (Coral). Notably, the FP32 DNN representation consumed even less energy than the C++ baseline, achieving 3.98 mJ on the QXSP-ML81 and 3.72 mJ on the Coral. In contrast, the FP16 implementation was slightly less efficient, requiring 4.00 and 5.34 mJ, respectively. Further reductions were achieved through INT8 quantization. On the QXSP-ML81, the INT8 implementation lowered the mean energy consumption to 2.01 mJ, while on the Coral board, it achieved 3.37 mJ. The most energy-efficient results were obtained using the hardware accelerators: the INT8 NPU implementation on the QXSP-ML81 reduced the energy use to 1.24 mJ, while the INT8 TPU implementation on the Coral achieved 1.67 mJ.

The mean energy showed a larger improvement in the higher-precision DNN representations than the load energy. The load energy for C++ was 0.80 mJ on the QXSP-ML81 and 1.30 mJ on the Coral. On the one hand, the FP32 and the FP16 representations increased the load consumption on the QXSP-ML81 to 1.15 and 1.16 MJ. On the other hand, the implementations reached 0.67 and 1.10 mJ on the Coral, representing a reduction compared to C++. The INT8 implementations then decreased the consumption for both hardware variants and for the implementation on the CPU and AI accelerators (see Figure 13a,b) compared to the corresponding C++ implementation.

The comparison of the mean energy consumption per inference for the classification showed that the Python code implementation had the highest mean energy consumption, with 15.73 mJ on the QSXP-ML81 and 35.54 mJ on the Coral. The C++ implementation reduced the mean energy consumption to 1.89 mJ on the QSXP-ML81 and 3.08 mJ on the Coral. The DNN FP32 reached 1.85 and 2.76 mJ and represents a further improvement to the C++ implementation. On the one hand, the DNN FP16 increased the energy consumption at the QSXP-ML81 to 1.93 mJ. On the other hand, on the Coral, it was nearly the same compared to the C++ implementation, with 2.98 mJ. The INT8 representation on the CPU Arm Cortex and the INT8 NPU and TPU versions made a large improvement. The INT8 implementation decreased the energy consumption to 0.45 mJ on the QSXP-ML81 and to 0.98 mJ on the Coral. When enabling the AI accelerator, the NPU reached an improvement to 0.12 mJ, and the TPU showed an improvement to 0.37 mJ.

Further comparisons with the load energy showed some trends for the classification task. The C++ implementation outperformed the high-precision DNN representation, with 0.24 mJ on the QXSP-ML81 and 0.35 mJ on the Coral board. The load energy for the FP32 and FP16 implementations represented an increase to 0.55 and 0.58 mJ on the QXSP-ML81 and 0.64 and 0.67 mJ on the Coral. The further quantization to INT8 improved the load energy to 0.11 and 0.22 mJ for the QXSP-ML81 and the Coral, respectively. The use of the AI accelerators then achieved a further improvement to 0.02 mJ for the NPU and 0.068 mJ for the TPU.

Similar to the inference time evaluation, Table 6 presents the relative energy consumption compared to the C++ implementation across various quantization levels of the DNN representations. The comparison included the mean energy consumption $W_{mean}$ and the load energy consumption $W_{load}$, expressed as percentage changes. The table highlights that nearly all the DNN representations demonstrated a reduction in the mean energy consumption. However, when considering the load energy, which excludes idle power consumption, significant improvements were primarily observed for the low-precision data types, such as INT8 and INT8 with hardware acceleration.

## 4. Discussion

This paper demonstrates a novel approach that allows for the implementation of the inferences of interpretable ML algorithms as DNNs on generic AI accelerators. The IDNNRep was tested with the Coral TPU and the QXSP-ML81 NPU. The benchmark tests performed in this paper demonstrate a significant inference time improvement compared to common implementations in C++ and especially in Python. First, this paper demonstrates the effect of quantization on the accuracy of the algorithms. On the one hand, a lower precision resulted in a higher prediction error for the algorithm. On the other hand, the quantization enabled, besides a reasonable drop in accuracy, the efficient implementation of these algorithms on edge hardware. In terms of numbers, the inference time was reduced by up to 94.0% using AI accelerators compared to the already efficient C++ implementation. Similarly, the DNN representations executed on the CPU led to an improvement in all the precision types. The FP32 version reduced the inference time by up to 65.9%, the FP16 version by 51.9%, and the INT8 version by 80.0% compared to the C++ implementation.

The comparison of the FP32 and FP16 showed that the FP32 inference outperformed the FP16 regarding the inference time. Since the FP16 models are reduced in memory and bandwidth requirements, they should normally execute faster than the FP32 models. This behavior could be triggered by the internal casting operation of TensorFlow Lite, which can slow down the inference process [58]. This internal casting process can occur if operations do not support the FP16 calculation, which leads to additional latency and memory effort. The non-availability of the delegate for FP16 operations leads to an FP32 inference with a casting overhead, which results in an increased inference time, even if the memory and bandwidth requirements are decreased. This internal casting overhead can be mitigated by employing mixed-precision models rather than full FP16 models, as mixed precision selectively uses FP16 where supported, while retaining FP32 for operations that lack efficient FP16 implementations, thereby reducing the inference time.

Besides the improvement in the inference time, this paper also demonstrates a significant reduction in the energy consumption. This metric was investigated using an application-based method, which depends on the hardware and operation modes, showing a significant decrease in the mean energy consumption of up to 93.6% with AI accelerators. Nearly all the quantization levels decreased the amount of consumed mean energy. The FP32 decreased the mean energy by up to 63.6%, the FP16 by up to 47.9%, and the INT8 by up to 76.3%. As for the inference time, FP32 outperformed FP16, although memory benefits were derived from the lower-precision networks. This was due to the high correlation between the inference time and energy: due to the similar current demand for both implementations, the increased inference time of the FP16 version also resulted in a higher energy consumption.

The load energy consumption resulted in a lower improvement than the mean energy consumption improvement. At the higher quantization level, the DNNs even resulted in a decrease in the load energy. The lower-quantization-level INT8 version still outperformed the common C++ implementation, and with the further usage of AI accelerators, this improvement increased to 90.8%. The results indicate that the inference time has a stronger impact on the mean energy consumption than on the load energy consumption. In comparison to the inference time, the current draw has a greater effect on the load energy than on the mean energy. The mean energy metric assumes that the energy usage between inferences was negligible, while the load energy metric accounts for idle energy consumption during those periods. This distinction enables a more precise evaluation of the implementation efficiency across different operating modes. Incorporating a sleep mode between inferences is recommended to further reduce the overall energy consumption. This strategy can significantly improve the mean energy usage and reduce idle-related overhead reflected in the load energy metric.

Although this study demonstrated a reduction in the inference time and the overall energy consumption, both critical metrics for system integration, it is essential to consider the specific application context of the algorithm. Suppose that the inference process constitutes only a small portion of the mean operational time. In that case, its impact on the energy efficiency may be relatively minor compared to the energy consumed during idle periods.

## 5. Conclusions and Future Work

This study explored the inference time and energy efficiency benefits of the IDNNRep for executing the interpretable ML algorithm inference as DNN representations on the edge hardware, including generic AI accelerators. This enables smart sensors to process data on the edge, reducing the latency and increasing the energy efficiency. With this novel approach, the volume of transmitted data over sensor networks is significantly diminished, shifting from continuous raw data streams to compact prediction outputs. The IDNNRep was demonstrated on an open-source AutoML toolbox. The proposed method outperformed conventional implementations of interpretable ML inference on edge devices in terms of both the inference time and the energy consumption, even without utilizing dedicated AI accelerators. When AI accelerators were employed, further improvements in both metrics were achieved. Deploying generic AI accelerators requires no additional effort once the DNN representation is created, enabling the interpretable ML inference to benefit from widely available hardware acceleration for DNNs, but this comes at a price of a reduced accuracy caused by the necessary quantization.

Optimizing the system’s operational modes, such as incorporating sleep modes, can yield further energy savings. The investigation in this paper was based on the comparative measurement of two selected hardware platforms. Due to hardware-dependent effects, the cross-comparison between these platforms was more complex and therefore not included in this study.

Future research could explore novel methods that further leverage the enhanced inference time and energy efficiency of quantized INT8 models. Systematic oversampling techniques may be applied to address the typical loss in the prediction accuracy associated with INT8 representations, e.g., by performing inference over multiple inputs to recover the lost accuracy.

An additional benefit that should be investigated in the future is the option offered by the IDNNRep to also train the interpretable ML algorithms on the edge hardware to allow for the influence of domain shifts to be reduced [23]. Compared to the conventional interpretable ML algorithms, which are limited to standardization and normalization techniques, this approach allows for the usage of transfer learning techniques, which have outperformed conventional methods in other studies [59]. This also includes different DNN-specific approaches, like federate learning.

## Figures and Tables

**Table 1:** List of the feature extraction, feature selection, classification, and regression algorithms of the AutoML toolbox, which are implemented as deep neural networks.

Processing Step	Methods
Feature Extraction	Adaptive linear approximation (ALA) [34]
	Best Daubechies wavelet (BDW) [35]
	Principal component analysis (PCA) [36]
	Statistical moment (StatMom) [22]
Feature Selection	Pearson correlation coefficient (Pearson) [37]
	RELIEFF [38]
	Recursive feature elimination support vector machine (RFESVM) [39]
	Spearman correlation coefficient (Spearman) [40]
Classification	Linear discriminant analysis with Mahalanobis distance classification (LDA-MD) [41,42]
Regression	Partial least squares regression (PLSR) [43]

**Table 2:** Description of the hydraulic system dataset.

Dataset	Observations	Signal Size	Task
HS (Valve)	1449	6000	Classification
HS (Acm)	1449	6000	Regression

**Table 3:** Measurement and source specification of the Keithley 2602B source meter.

	Range	Accuracy
Voltage Source Specification	0 ... 6 V	0.02%±1.8 mV
Current Measurement Specification	0 ... 3 A	0.05%±3.5 mA

**Table 4:** Accuracy comparison of regression and classification task for the different precision levels.

Task	Accuracy: FP32 Python, C++, DNN	Accuracy: FP16	Accuracy: INT8
Regression	91.8% (**RMSE**: 10.6 bar)	90.4% (**RMSE**: 12.5 bar)	85.9% (**RMSE**: 18.3 bar)
Classification	99.9%	99.8%	99.4%

**Table 5:** Inference time comparison of the IDNNRep relative to the C++ implementations. Positive values indicate an increased inference time, while negative values indicate a reduced inference time compared to the C++ implementations. The percentage was calculated by the accurate values.

	Regression [%]		Classification [%]	
	QXSP-ML81	Coral	QXSP-ML81	Coral
**FP32**	−40.8	−65.9	−22.9	−22.3
**FP16**	−40.0	−51.9	−19.5	−15.3
**INT8**	−69.9	−68.3	−80.0	−72.1
**INT8 AI ACC**	−81.5	−84.6	−94.0	−88.7

**Table 6:** Energy consumption comparison of the DNN representations relative to the C++ implementations. Positive values indicate an increased energy consumption, while negative values indicate a reduced energy consumption compared to the C++ implementations.

	Regression [%]				Classification [%]			
	QXSP-ML81		Coral		QXSP-ML81		Coral	
	$W_{mean}$	$W_{load}$	$W_{mean}$	$W_{load}$	$W_{mean}$	$W_{load}$	$W_{mean}$	$W_{load}$
FP32	−28.6	+43.6	−63.6	−47.0	−2.2	+127.2	−10.3	+84.2
FP16	−27.6	+45.4	−47.9	−18.8	−2.1	+137.0	−3.4	+91.4
INT8	−67.1	−50.8	−67.1	−58.9	−76.3	−53.5	−68.1	−36.8
INT8 AI ACC	−82.0	−85.6	−83.7	−76.8	−93.6	−90.9	−88.0	−80.5

## Data Availability

All the datasets used in this study are publicly available. Detailed information on the sources of these datasets can be found in the corresponding sections of this paper.

## Abbreviations

The following abbreviations are used in this manuscript:

AI	Artificial Intelligence
ALA	Adaptive Linear Approximation
Acm	Accumulator (Hydraulic)
ASIC	Application-Specific Integrated Circuit
BDW	Best Daubechies Wavelet
C/R	Classification and Regression
CPU	Central Processing Unit
DLF	Deep Learning Framework
DNN	Deep Neural Network
DUT	Device under Test
FE	Feature Extraction
FESC/R	Feature Extraction, Feature Selection, and Regression or Classification
FP16	Floating Point 16
FP32	Floating Point 32
FPGA	Field-Programmable Gate Array
FS	Feature Selection
GM	Gas Measurement
GPU	Graphical Processing Unit
HS	Hydraulic System
IDNNRep	Interpretable Deep Neural Network Representation
INT8	Integer 8
LDA-MD	Linear Discriminant Analysis with Mahalanobis Distance Classification
ML	Machine Learning
NPU	Neural Processing Unit
ONNX	Open Neural Network Exchange
PCA	Principal Component Analysis
Pearson	Pearson Correlation Coefficient
PLSR	Partial Least Squares Regression
PM	Predictive Maintenance
PTQ	Post-Training Quantization
RFESVM	Recursive Feature Elimination Support Vector Machine
RMSE	Root Mean Square Error
SHM	Structural Health Monitoring
SM	Source Meter
SMU	Source Measure Unit
SoC	System-on-Chip
Spearman	Spearman Correlation Coefficient
StatMom	Statistical Moment
TL	Transfer Learning
TPU	Tensor Processing Unit
